# Pulse wave velocity does not predict ventricular strain in Ross patients

**DOI:** 10.1186/1532-429X-16-S1-P113

**Published:** 2014-01-16

**Authors:** Jason Christensen, Jimmy C Lu, Sunkyung Yu, Janet Donohue, Prachi P Agarwal, Maryam Ghadimi Mahani, Adam L Dorfman

**Affiliations:** 1 Division of Pediatric Cardiology, Department of Pediatrics and Communicable Diseases, University of Michigan, C.S. Mott Children's Hospital, Ann Arbor, Michigan, USA; 2 Section of Pediatric Radiology, Department of Radiology, University of Michigan, Ann Arbor, Michigan, USA; 3 Division of Cardiothoracic Radiology, Department of Radiology, University of Michigan, Ann Arbor, Michigan, USA

## Background

Worsened aortic pathology may adversely affect ventricular function over time. Patients requiring the Ross procedure are at risk for aortopathy including worsened aortic stiffness, and are at risk for left ventricular (LV) dysfunction. Early changes in vascular stiffness can be measured with cardiovascular magnetic resonance imaging (CMR) by pulse wave velocity (PWV). Likewise, decreased LV strain may precede changes in ejection fraction. We hypothesized thoracic aorta PWV would predict impaired left ventricular systolic function in patients after the Ross procedure.

## Methods

Ross patients studied by CMR from 2007-2013 at the University of Michigan Health System were reviewed. Exclusion criteria included CMR under sedation/anesthesia, neoaortic valve replacement, or inadequate CMR data. PWV was calculated with the aortic arch distance between ascending and descending aorta on phase contrast images and the time between the pulse wave feet at those sites. Peak LV circumferential strain was measured using feature tracking software (TomTec, Unterschleissheim, Germany). Correlation of PWV and LV systolic strain with LV systolic parameters and other variables was examined via Pearson and Spearman correlation coefficients, two-sided t-test, analysis of variance and Wilcoxon rank sum test where appropriate.

## Results

31 patients were included, with median age at CMR of 28.1 years (range 15.4-61.9) and mean time from Ross procedure of 11.5 ± 3.9 years. Worsened PWV was associated with prior root replacement (7.2 m/s(5.4-8.7) vs. 4.0 m/s(2.7-5.1), p = 0.05) and aortic insufficiency (r = 0.47, p = 0.01), and correlated with aortic dilatation at the sinotubular junction (r = 0.53, p = 0.01) and the ascending aorta (r = 0.49, p = 0.01), excluding patients with root replacement. LV circumferential strain was associated with measures of LV systolic function: ejection fraction (r = -0.77, p = < .0001), end diastolic volume (r = 0.62, p = 0.0002), end systolic volume (r = 0.73, p = < .0001) and mass (r = 0.62, p = 0.0002). It was also associated with systemic hypertension (-19.1 ± 2.9 vs. -24.1 ± 3.7, p = 0.01) and pre-Ross diagnosis of aortic insufficiency versus aortic stenosis (-20.7 ± 4.4 vs. -27.0 ± 3.8, p = 0.03). There was no correlation between PWV and measurements of LV systolic performance, including strain (r = 0.09, p = 0.61), ejection fraction (r = 0.12, p = 0.52) or end systolic volume (r = 0.13, p = 0.50).

## Conclusions

Patients following Ross procedure have increased aortic stiffness associated with aortic root dilatation and history of aortic root replacement subsequent to their Ross operation. While systemic hypertension and pre-operative diagnosis of aortic insufficiency were correlated with worsened LV circumferential strain, this study could not detect a relationship between aortic stiffness and global measures of LV systolic function.

## Funding

None.

